# Early life stress induces attention-deficit hyperactivity disorder (ADHD)-like behavioral and brain metabolic dysfunctions: functional imaging of methylphenidate treatment in a novel rodent model

**DOI:** 10.1007/s00429-016-1244-7

**Published:** 2016-06-16

**Authors:** J. Bock, S. Breuer, G. Poeggel, K. Braun

**Affiliations:** 1 Institute of Biology, Department of Zoology/Developmental Neurobiology, Otto von Guericke University, Leipziger Str. 44, 39118 Magdeburg, Germany; 2 Center for Behavioral Brain Science, Otto von Guericke University Magdeburg, Magdeburg, Germany; 3 Institute for Biology, Human Biology, University of Leipzig, 04103 Leipzig, Germany

**Keywords:** Limbic, Prefrontal cortex, Childhood adversity, Stress, Dopamine

## Abstract

In a novel animal model *Octodon degus* we tested the hypothesis that, in addition to genetic predisposition, early life stress (ELS) contributes to the etiology of attention-deficit hyperactivity disorder-like behavioral symptoms and the associated brain functional deficits. Since previous neurochemical observations revealed that early life stress impairs dopaminergic functions, we predicted that these symptoms can be normalized by treatment with methylphenidate. In line with our hypothesis, the behavioral analysis revealed that repeated ELS induced locomotor hyperactivity and reduced attention towards an emotionally relevant acoustic stimulus. Functional imaging using (^14^C)-2-fluoro-deoxyglucose-autoradiography revealed that the behavioral symptoms are paralleled by metabolic hypoactivity of prefrontal, mesolimbic and subcortical brain areas. Finally, the pharmacological intervention provided further evidence that the behavioral and metabolic dysfunctions are due to impaired dopaminergic neurotransmission. Elevating dopamine in ELS animals by methylphenidate normalized locomotor hyperactivity and attention-deficit and ameliorated brain metabolic hypoactivity in a dose-dependent manner.

## Introduction

Attention-deficit hyperactivity disorder (ADHD) is a heterogeneous syndrome, which is characterized by behavioral and cognitive dysfunctions, including inattention, impulsivity and hyperactivity (Kirby et al. 2002; Krain and Castellanos 2006; Pasini et al. 2007; Goldman et al. 1998). Clinical studies have emphasized a genetic predisposition; however, more recently there is evidence that adverse environmental factors, such as chronic family conflicts, decreased family cohesion and parental psychopathology also may play a significant role (Biederman et al. 1995; Counts et al. 2005; Miller et al. 2006). The vast majority of animal models, in which the mechanisms and etiology of attention-deficit hyperactivity disorder (ADHD) are investigated experimentally, such as the spontaneously hypertensive rat (Sagvolden 2000), the dopamine transporter knockout mouse (Gainetdinov and Caron 2001), and others (Davids et al. 2003), are based on the analysis of genetic predispositions. In contrast, the contribution of environmental factors on the etiology of ADHD has been studied in much less detail. The semi-precocial rodent *Octodon degus* represents an ideal animal model for the analysis of the environmental, experience-mediated influences, since degus are—similar to human babies and unlike the commonly used altricial laboratory rodents—born with functional sensory systems (open ears and eyes). Previous studies in this animal model have shown that repeated disturbance of the family environment during the first weeks of life results in behavioral dysfunctions resembling symptoms of ADHD (Braun et al. 2003). These behavioral abnormalities are paralleled by changes of dendritic spine density in prefrontal and limbic brain areas (Helmeke et al. 2001; Poeggel et al. 2003a), which can be normalized by a chronic treatment with methylphenidate (Zehle et al. 2007), a compound that is used to treat ADHD symptoms in children and adolescents.

The aim of this study was to test the hypothesis that environmental factors such as early life adversity contributes to the etiology of ADHD-like behavioral symptoms, and results in dysfunctional activation patterns in limbic and prefrontal regions. Based on previous observations that ELS significantly alters dopaminergic innervation patterns in the medial prefrontal cortex and in the nucleus accumbens (Braun et al. 2000; Gos et al. 2006; Kunzler et al. 2015) and reduces dopaminergic functions (Jezierski et al. 2007), we predicted that treatment with methylphenidate should “normalize” the ELS-induced ADHD-like behavioral and brain dysfunctions.

## Materials and methods

### Experimental subjects

*Octodon degus* (“trumped tailed rat”) were bred in our animal facility and housed in wire cages (l/w/h 50 cm × 42 cm × 67 cm) in air-conditioned rooms (22 °C) under a 12/12 h light/dark cycle. Drinking water, rat diet pellets and vegetables were available ad libitum. Families consisted of an adult couple and their offspring. All experiments were performed in accordance with the European Community’s Council Directive of 24 Nov 1986 (86/609/EEC), the experimental protocols were approved by the ethics committee of the government of the state of Saxony-Anhalt according to the German guidelines for the care and use of animals in laboratory research (§8, Abs. 1, 25.05.1998).

### Experimental design (Fig. 1)

### Rearing conditions

Early life stress group (ELS): pups were exposed to daily parental separation from postnatal day (PND) 1 until PND 21. During the separation period (1 h per day) the animals were removed from their parents and siblings and individually placed in small opaque isolation cages (size: 39 × 10 × 10 cm) with fresh bedding. Acoustic and olfactory but no direct social contact between the siblings was possible.

**Fig. 1:**
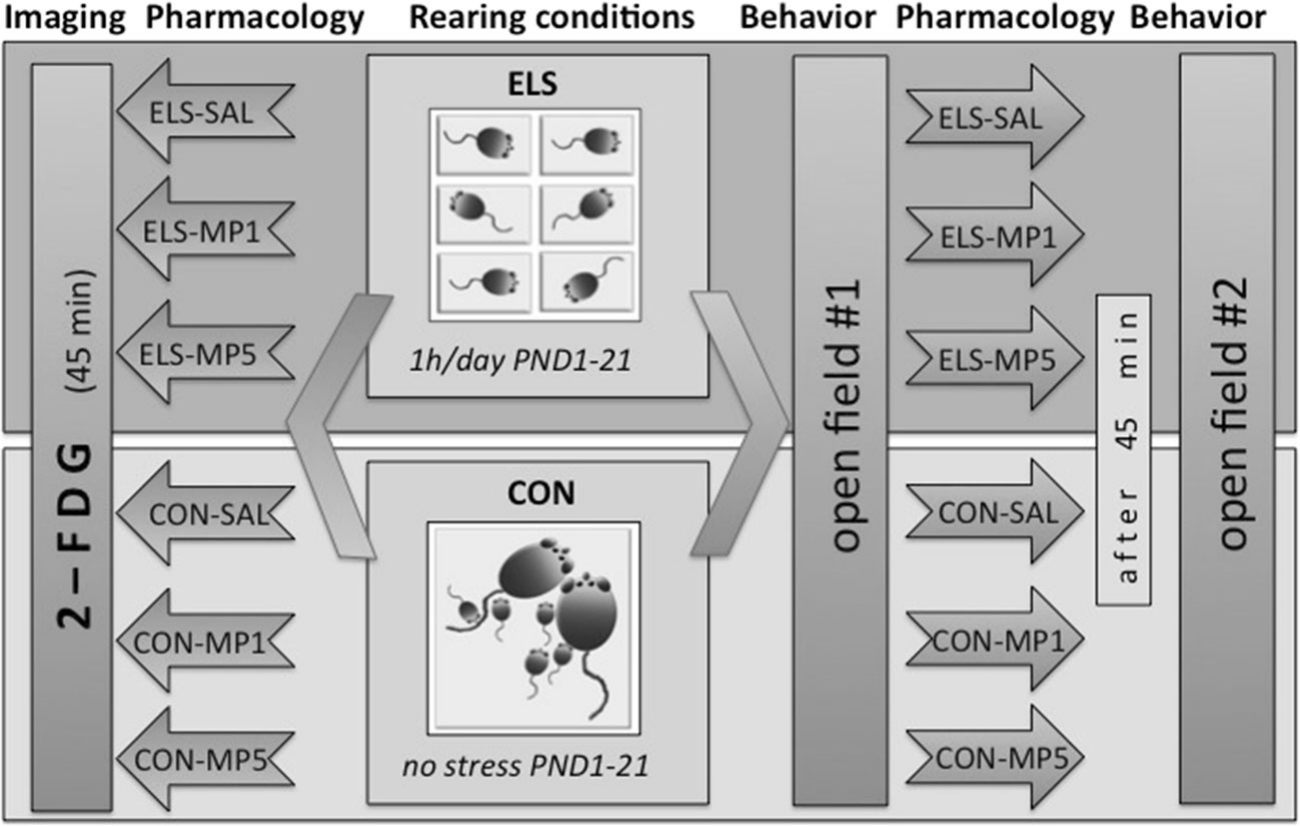
Experimental design, note that animals for behavioral and 2FDG experiments are from different cohorts

Control group (CON): pups of this group remained undisturbed in their home cages with their family until PND 21.

### Behavioral analysis

#### Locomotor activity (exploratory behavior and impulsivity)

The animals of the ELS and CON groups and the pharmacological treatment groups (see below) were tested in a standard open field (OF) test. The size of the OF was 80 × 80 × 40 cm, with opaque dark-gray plastic walls and floor. The open field arena was subdivided into a center part, which had the same area size as the peripheral region along the walls. The animals were individually placed in the open field for 5 min/test and the behavior was recorded using a video tracking system (EthoVision®, NOLDUS, Wageningen, The Netherlands), delivering data sets for the following parameters:

- Running activity: (center plus periphery of the OF) was measured as distance (m) covered by each animal during the 5-min OF test.
- Center exploration was defined as horizontal activity in the center area of the open field during the 5-min test and calculated as ratio of the running distance (m) covered in the center area relative to the total OF running distance.
- Rearing was measured by counting the numbers of vertical rearing movements.
- Self-grooming, i.e., the number of grooming activities on various body parts including back, head, face, nose, ears, etc.

#### Attentiveness

**Fig. 2:**
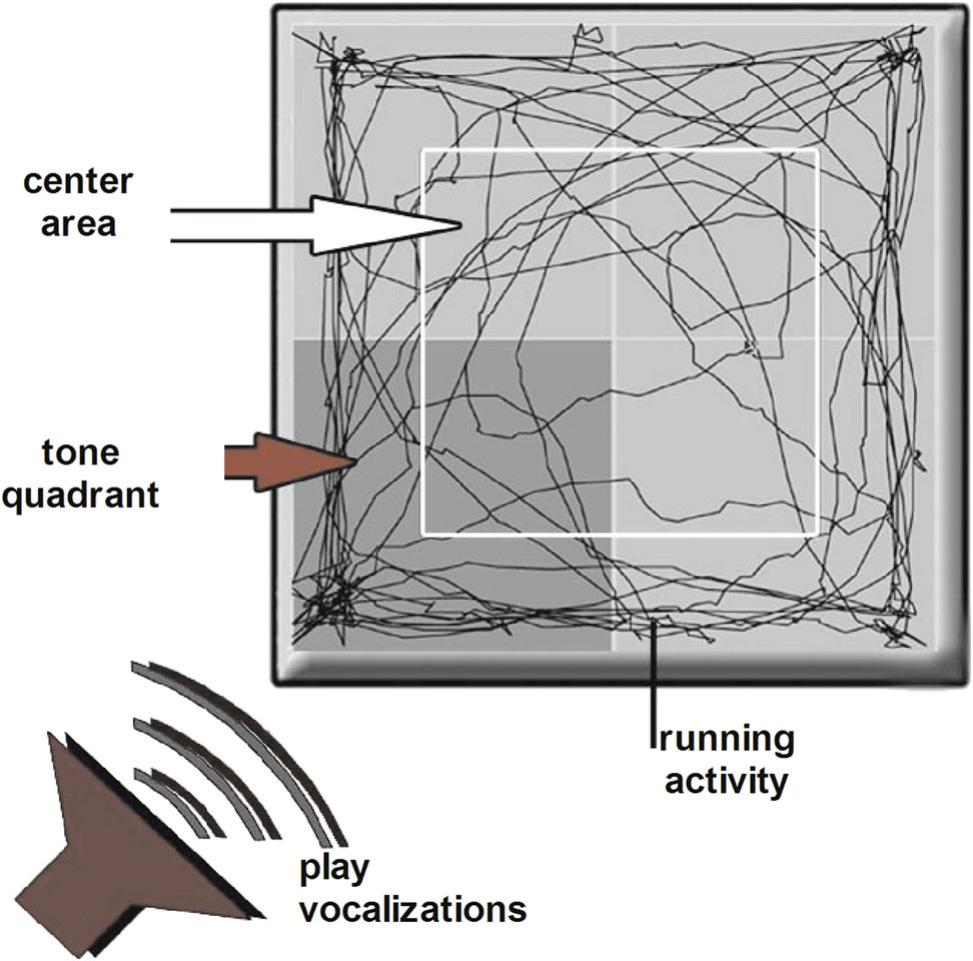
Behavioral analysis of attentiveness using a modified open field arena. A loudspeaker in one corner emitted play vocalizations from siblings

Animals of the ELS and CON groups and the pharmacological treatment groups (see below) were tested in a modified OF test (Fig. 2) that allowed quantitative analysis of the responsiveness (“attentiveness”) towards an acoustic emotionally relevant stimulus (play and contact vocalizations emitted from young degus). The open field arena was subdivided into 4 quadrants of same area size. From one corner of the arena degu vocalizations were presented from a loudspeaker, which was not visible to the test animal, the respective quadrant was defined as tone quadrant. The animals were individually placed in the open field for 5 min/test and the following parameters were assessed.

- Running activity in the tone quadrant (% of total running activity)
- Time spent (s) in the tone quadrant
- Vertical activity: see above
- Self-grooming: see above

### Pharmacological intervention

#### Standard open field test #1: effects of ELS on exploratory and impulsive behavior

On PND 22, the behavioral parameters outlined above were compared in pharmacologically untreated unstressed CON (*n* = 29) and ELS animals (*n* = 30) as described above.

#### Standard open field test #2: effects of methylphenidate on exploratory and impulsive behavior in ELS and CON animals

The animals of stressed (ELS) and control (CON) litters were randomly assigned to the following pharmacological treatment groups (Fig. 1); a split litter design was applied to avoid litter effects.

- Saline-treated ELS animals (ELS-SAL, *n* = 11) and saline-treated unstressed controls (CON-SAL, *n* = 13).
- 1 mg/kg methylphenidate-treated ELS animals (ELS-MP1, *n* = 9) and 1 mg/kg methylphenidate-treated unstressed controls (CON-MP1, *n* = 8).
- 5 mg/kg methylphenidate-treated ELS animals (ELS-MP5, *n* = 10) and 5 mg/kg methylphenidate-treated unstressed controls (CON-MP5, *n* = 8).

#### Modified open field test #1: effects of ELS on attentiveness

On PND 22, the behavioral parameters outlined above were compared in pharmacologically untreated unstressed CON (*n* = 24) and ELS animals (*n* = 25) in the modified open field test as described above (Helmeke et al. 1998; Poeggel et al. 2003a; Helmeke et al. 2001).

#### Modified open field test #2: effects of methylphenidate on attentiveness in ELS and CON animals

The animals of stressed (ELS) and control (CON) litters were randomly assigned to the following pharmacological treatment groups (Fig. 1), a split litter design was applied to avoid litter effects.

- Saline-treated ELS animals (ELS-SAL, *n* = 8) and saline-treated unstressed controls (CON-SAL, *n* = 8).
- 1 mg/kg methylphenidate-treated ELS animals (ELS-MP1, *n* = 9) and 1 mg/kg methylphenidate-treated unstressed controls (CON-MP1, *n* = 8).
- 5 mg/kg methylphenidate-treated ELS animals (ELS-MP5, *n* = 8) and 5 mg/kg methylphenidate-treated unstressed controls (CON-MP5, *n* = 5).

For the behavioral analysis during the respective open field test #2 the animals received an intraperitoneal (i.p.) injection of either 0.2 ml sterile saline, 1 mg/kg methylphenidate (MP1) or 5 mg/kg methyphenidate (MP5) solution (MP: Sigma-Aldrich, Steinheim, Germany), respectively, which was applied directly after open field test #1. After the injection the animals were returned to the home cage for 45 min and then individually exposed to open field test #2. Between each test the open field was wiped with 2 % acidic acid. The daily testing routine was balanced across the experimental groups.

### Statistical analysis

#### Open field test #1: effects of ELS on exploratory, impulsive behavior and attentiveness

For the described parameters, the effect of rearing condition (REAR) was tested using Student’s *t* test with a significance level set at *p* < 0.05, (GraphPad Prism version 5.00, GraphPad Software, San Diego, CA, USA).

#### Open field test #2: effects of methylphenidate on exploratory, impulsive behavior and attentiveness

The effects of the different MP doses on the two rearing conditions were analyzed using a two-way ANOVA with the factors rearing condition (REAR) and pharmacological treatment (PHARM). Since the aim of this study was to test the “therapeutic” effects of MP treatment the values of the CON-SAL control group were defined as baseline to which the data of all experimental groups were referred using a one-sample *t* test as post hoc test. The level of significance was set to *p* ≤ 0.05 (GraphPad Prism version 5.00, GraphPad Software, San Diego, CA, USA).

### Functional imaging

#### 2-FDG-autoradiography

On PND 22, males of stressed (ELS) and control (CON) litters were randomly assigned to the following pharmacological treatment groups (Fig. 1).

- Saline-treated early stressed animals (ELS-SAL, *n* = 5) and saline-treated unstressed controls (CON-SAL, *n* = 5).
- 1 mg/kg MP-treated stressed animals (ELS-MP1, *n* = 5) and 1 mg/kg MP-treated unstressed controls (CON-MP1, *n* = 5).
- 5 mg/kg MP-treated stressed animals (ELS-MP5, *n* = 5) and 5 mg/kg MP-treated unstressed controls (CON-MP5, *n* = 5).

**Fig. 3:**
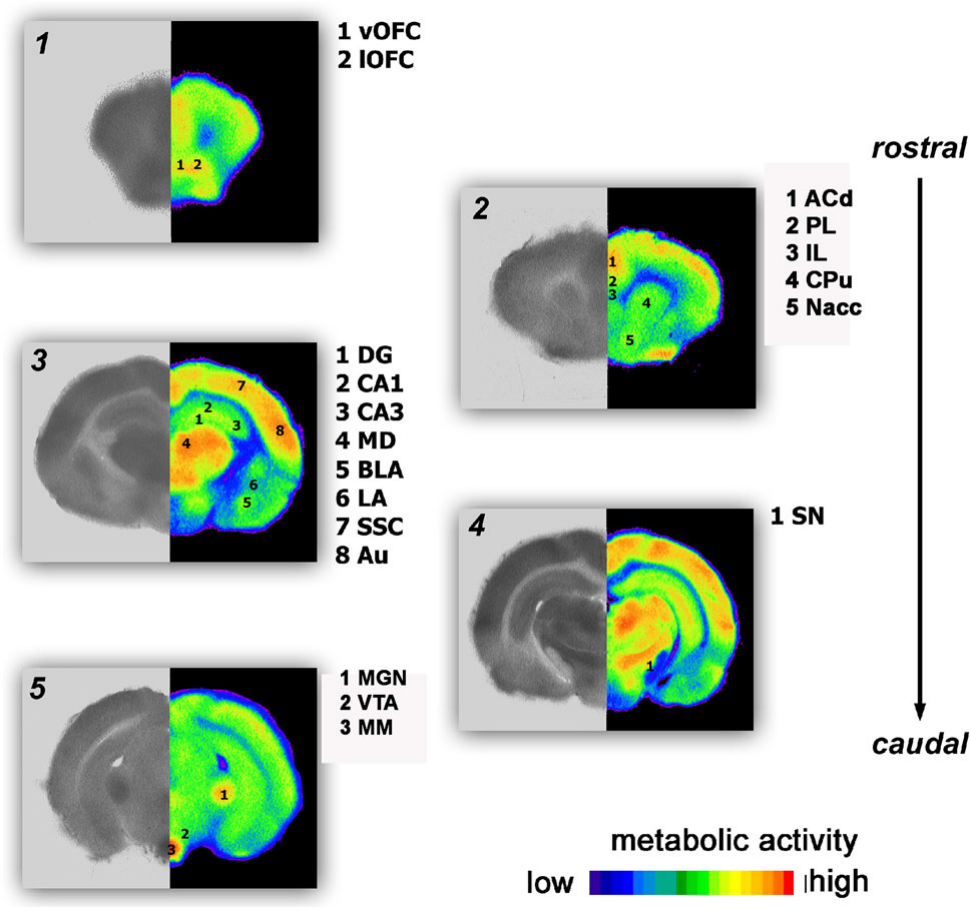
Brain areas, quantitatively analyzed in the 2FDG experiment: infralimbic cortex (IL), prelimbic cortex (PL), ventral (vOFC) and lateral orbitofrontal cortex (lOFC), anterior cingulate cortex (ACd), basolateral amygdala (BLA), lateral amygdala (LA), medio-dorsal thalamus (MD), auditory cortex (Au), caudate putamen (CPu), dentate gyrus (DG), hippocampal subregions CA1, CA3, medial geniculate nucleus (MGN), mammillary nuclei (MM), nucleus accumbens (Nacc), substantia nigra (SN), somatosensory cortex (SSC)

Male siblings of CON or ELS litters were placed in an opaque experimental chamber. After 10 min habituation each animal received an i.p. injection of 0.2 ml of a mixture of 9 µCi 2-fluoro-2-deoxy-d-glucose [14C(U)] 2-FDG (American Radiolabeled Chemicals) in sterile 0.9 % saline with either 1 or 5 mg/kg MP solution or without MP, respectively. After 45 min the animals were decapitated, their brains quickly removed and rapidly frozen. 40-µm frozen frontal sections were mounted on glass slides, immediately dried at a temperature of 40 °C on a heating plate and exposed on X-ray Film (Kodak Biomax MR). Selected regions of interest (ROI) were densitometrically quantified using NIH Image software. 2-FDG uptake was calculated for each ROI as relative value to the mean gray value of the corpus callosum, which served as internal control. The following brain regions (Fig. 3) were quantitatively analyzed: infralimbic cortex (IL), prelimbic cortex (PL), ventral (vOFC) and lateral orbitofrontal cortex (lOFC), anterior cingulate cortex (ACd), basolateral amygdala (BLA), lateral amygdala (LA), medio-dorsal thalamus (MD), auditory cortex (Au), caudate putamen (CPu), dentate gyrus (DG), hippocampal subregions CA1, CA3, medial geniculate nucleus (MGN), mammillary nuclei (MM), nucleus accumbens (Nacc), substantia nigra (SN), and somatosensory cortex (SSC).

### Statistical analysis

Since no hemispheric differences were found, the values for the hemispheres were pooled and a two-way ANOVA with the factors rearing condition (REAR) and pharmacological treatment (PHARM) was applied. Again the values of the CON-SAL control group were defined as baseline to which the data of all experimental groups were referred using a one-sample *t* test as post hoc test. The level of significance was set to *p* ≤ 0.05.

### Preparation of figures

Digital images were processed using Adobe Photoshop 7.0 (Adobe Systems Incorporated, USA) and assembled into montages. Only general adjustments of color, contrast, and brightness were made.

## Results

### Behavioral analysis

#### Exploratory activity in the standard open field

##### Test #1: ELS increases exploratory motor activity and impulsivity

**Fig. 4:**
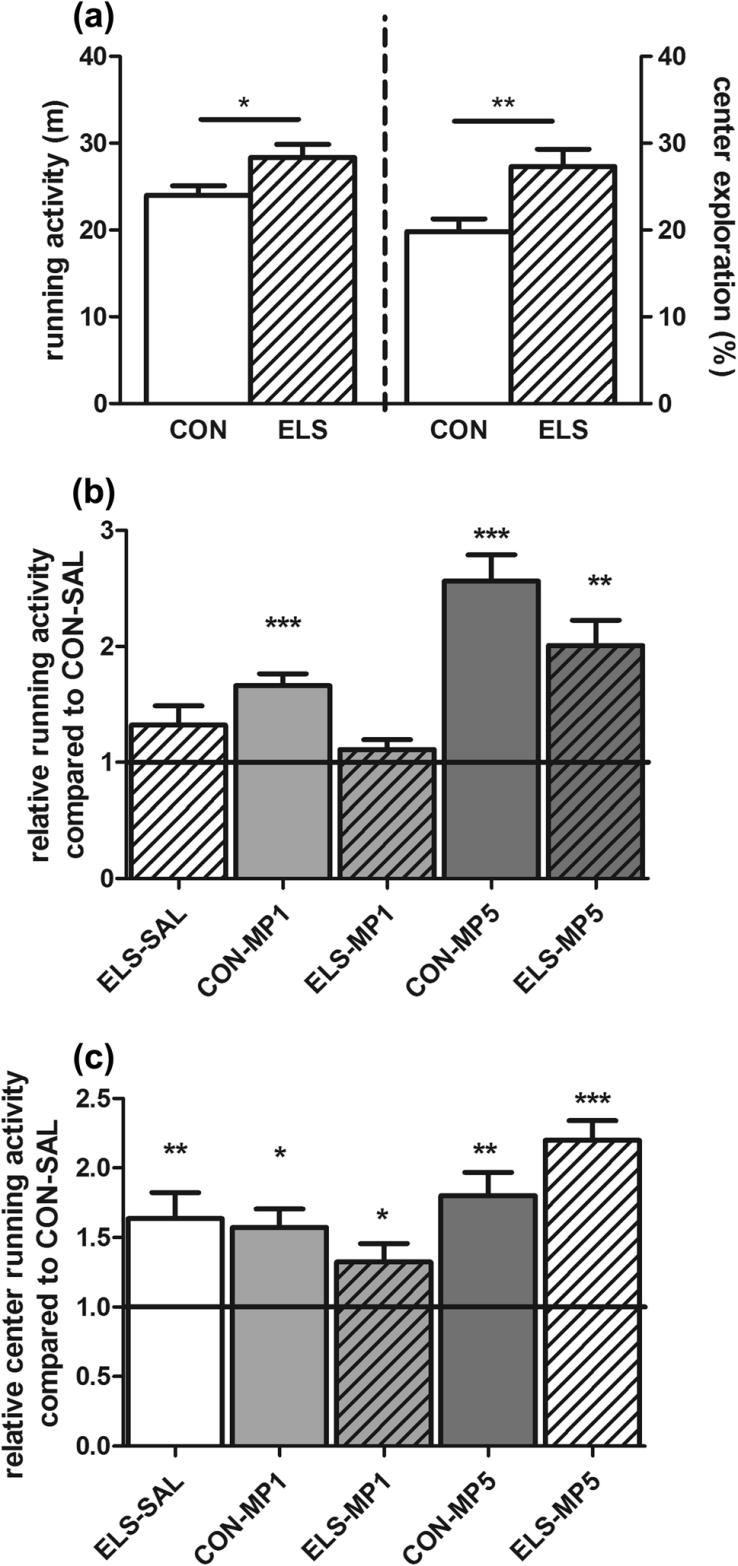
Effect of ELS and pharmacological treatment with methylphenidate (MP) on motor activity in the standard open field. Open field test #1: **a** effects of ELS on running activity in the total arena (*left Y-axis*) and relative running activity in the center of the arena (*right Y-axis*). Open field test #2: **b** effect of MP on total running activity in relation to the CON-SAL group (*black line*). **c** Effect of MP on center running activity related to the CON-SAL group (*black line*)

*t* Test revealed a significant increase of total running activity (*p* = 0.024) and running activity in the center of the OF arena (*p* = 0.004) in the ELS group compared to the CON group (Fig. 4a). Rearing and grooming did not differ between the two groups (data not shown).

##### Test #2: methylphenidate “normalizes” running activity and differentially affects ELS and CON animals

Total running activity: two-way ANOVA revealed significant main effects for the factor PHARM (*F*_2,53_ = 27.33, *p* < 0.0001) as well as a significant interaction of REAR × PHARM (*F*_2,53_ = 5.401, *p* = 0.007). Post hoc one-sample *t* test revealed that treatment of ELS animals with 1 mg/kg MP decreased running activity down to the level of the CON-SAL group (“therapeutic” effect), whereas 5 mg/kg MP significantly increased running activity in the ELS group above the level of the CON-SAL group (*p* < 0.01) (Fig. 4b). Treatment of CON animals with 1 mg/kg MP (*p* < 0.001) or 5 mg/kg MP (*p* < 0.001) increased running activity compared to the CON-SAL group (Fig. 4b).

Center activity: two-way ANOVA revealed significant main effects for the factor REAR (*F*_1,53_ = 4.675, *p* = 0.035), PHARM (*F*_2,53_ = 11.99, *p* < 0.0001) and a significant interaction of REAR × PHARM (*F*_2,53_ = 4.754, *p* = 0.0126). Post hoc test revealed that ELS-SAL animals displayed significantly higher center activity compared to CON-SAL animals (*p* < 0.01) (Fig. 4c). However, no “therapeutic” normalization of center activity was observed after treatment of ELS animals with 1 mg/kg MP, since these animals remained more active in the center compared to the CON-SAL group (*p* < 0.05). Treatment of ELS animals with 5 mg/kg MP further increased center activity (*p* < 0.001, Fig. 2c) compared to the CON-SAL group. In addition, treatment of CON animals with 1 mg/kg MP (*p* < 0.05) or 5 mg/kg MP (*p* < 0.01) increased center activity compared to the CON-SAL group (Fig. 4c).

Rearing activity: two-way ANOVA revealed significant main effects for the factor PHARM (*F*_2,53_ = 6.023, *p* = 0.0095). Post hoc test revealed significantly decreased rearing activity in the CON-MP5 group compared to the CON-SAL group (*p* < 0.05, data not shown).

Grooming behavior: two-way ANOVA revealed significant main effects for the factor REAR (*F*_1,53_ = 7.244, *p* < 0.0001). Post hoc test revealed significantly decreased grooming activity in the CON-MP5 group compared to the CON-SAL group (*p* < 0.05, data not shown).

#### Attentiveness in the modified open field

##### Test #1: ELS reduces attentiveness and increases grooming and rearing behavior in ELS animals

**Fig. 5:**
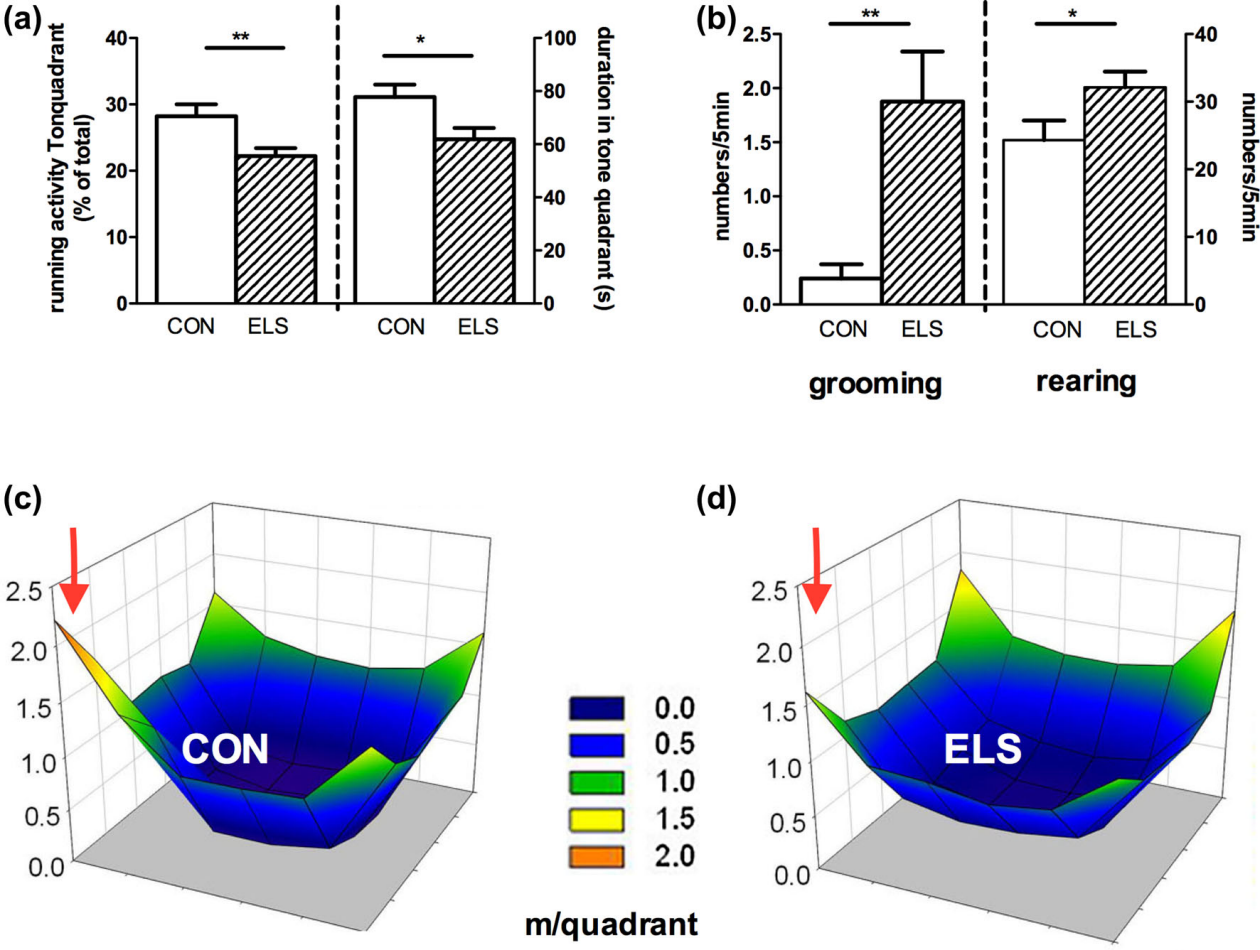
Effect of ELS on attentiveness in the modified open field. Open field test #1: **a** Running activity in the tone quadrant (% of total running activity, *left Y-axis*) and time, spent in the tone quadrant (in seconds, *right Y-axis*). **b** Self-grooming (*left*) and rearing (*right*) activity. Graphic illustration of the running activities in the modified open field arena in CON (**c**) and ELS animals (**d**); *red arrow* indicates the tone quadrant

*t* Test revealed a significant reduction of running activity and time spent in the tone quadrant in the ELS group compared to the CON group (running activity: *p* = 0.008, time spent: *p* = 0.0163) (Fig. 5a, c, d). In contrast, the parameters rearing and grooming were increased in the ELS animals compared to the CON animals (*p* = 0.042, grooming: 0.0012) (Fig. 5b).

##### Test #2: methylphenidate treatment “normalizes” attentiveness and grooming behavior in a dose-dependent manner

**Fig. 6:**
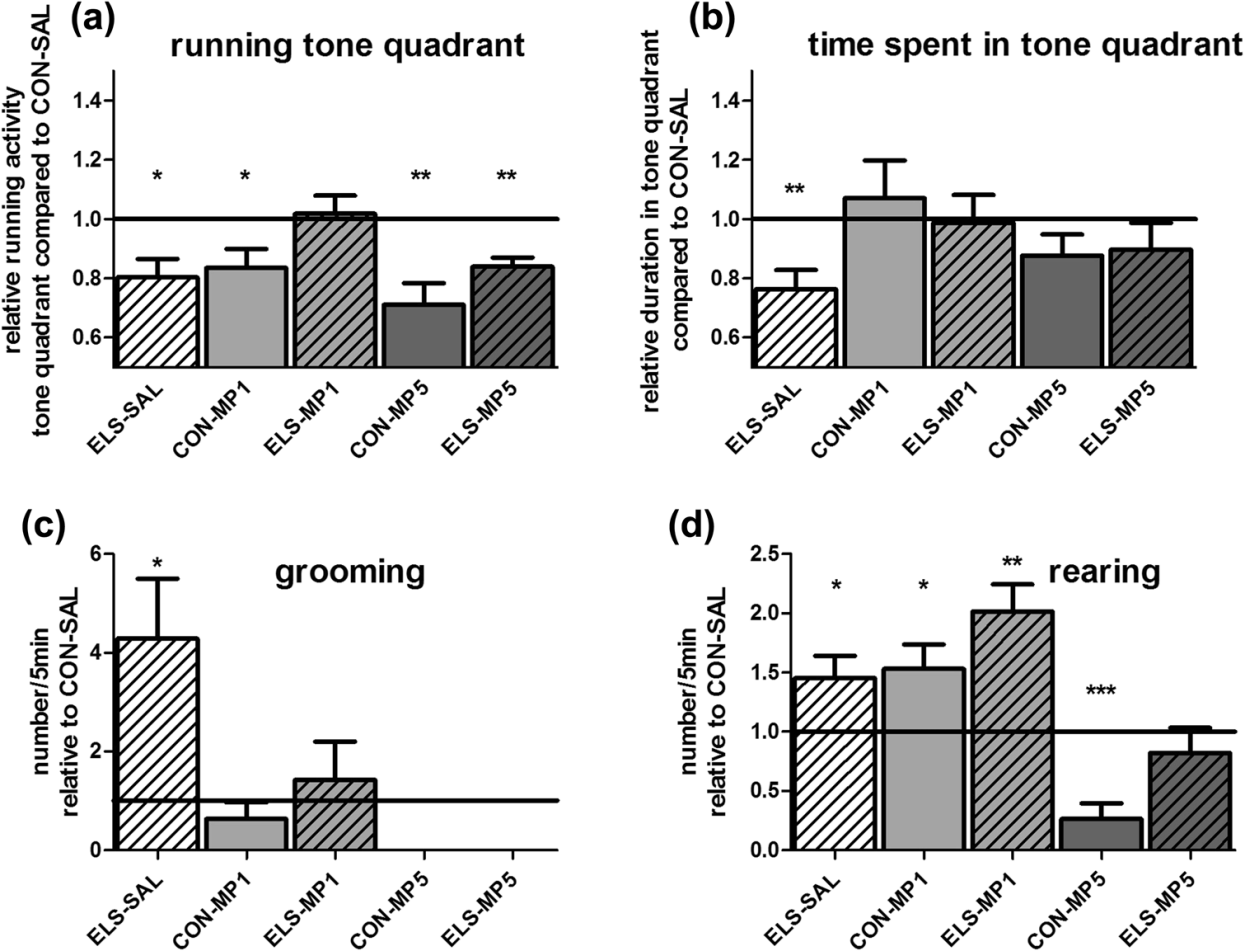
Effects of MP treatment on attentiveness in the modified open field. Open field test #2: **a** effect of MP on running activity in the tone quadrant related to the CON-SAL group (*black line*). **b** Effect of MP on the time spent in the tone quadrant related to the CON-SAL group (*black line*). **c** Effect of MP on self-grooming related to the CON-SAL group (*black line*). **d** Effect of MP on rearing related to the CON-SAL group (*black line*)

Running activity and time spent in the tone quadrant: two-way ANOVA revealed a significant interaction of REAR × PHARM (*F*_2,43_ = 4.292, *p* = 0.02) for running activity. Similar to the non-injected ELS group (see above Fig. 5), the ELS-SAL group displayed significantly lower running activity in the tone quadrant compared to the CON-SAL group (*p* < 0.05) (Fig. 6a). Similarly, time spent in the tone quadrant was significantly lower in the ELS-SAL group compared to the CON-SAL group (*p* < 0.01) (Fig. 6b). Treatment of the ELS group with 1 mg/kg MP “normalized” running activity as well as time spent in the tone quadrant to the level of the CON-SAL group (Fig. 6a, b).

In contrast, treatment of ELS animals with 5 mg/kg MP had no therapeutic effect on relative running activity since these animals displayed a decrease of this parameter (*p* < 0.01) compared to the CON-SAL group (Fig. 6a). However, a partial normalization with this dose was seen for the time spent in the tone quadrant (Fig. 6b).

Grooming behavior: two-way ANOVA revealed significant main effects for the factor PHARM (*F*_2,43_ = 7.918, *p* = 0.0012), the factor REAR (*F*_1,43_ = 6.302, *p* = 0.0159) and the interaction of REAR × PHARM (*F*_2,43_ = 3.288, *p* = 0.0469). Similar to the non-injected CON and ELS groups (see above Fig. 5b), the SAL-injected ELS animals showed increased grooming (*p* < 0.05) behavior (Fig. 6c). Grooming behavior was completely “normalized” in the ELS group by treatment with 1 mg/kg MP, whereas 5 mg/kg MP completely abolished grooming behavior (Fig. 6c).

Rearing activity: two-way ANOVA revealed significant main effects for the factors PHARM (*F*_2,43_ = 17.52, *p* < 0.0001) and REAR (*F*_1,43_ = 8.476, *p* = 0.0057). Similar to the non-injected CON and ELS groups (Fig. 5b), the ELS-SAL animals showed increased rearing (*p* < 0.05) behavior (Fig. 6d). The dose of 5 mg/kg MP reduced rearing behavior of ELS animals down to the level of the CON-SAL group (Fig. 6d).

### Functional Imaging

**Fig. 7:**
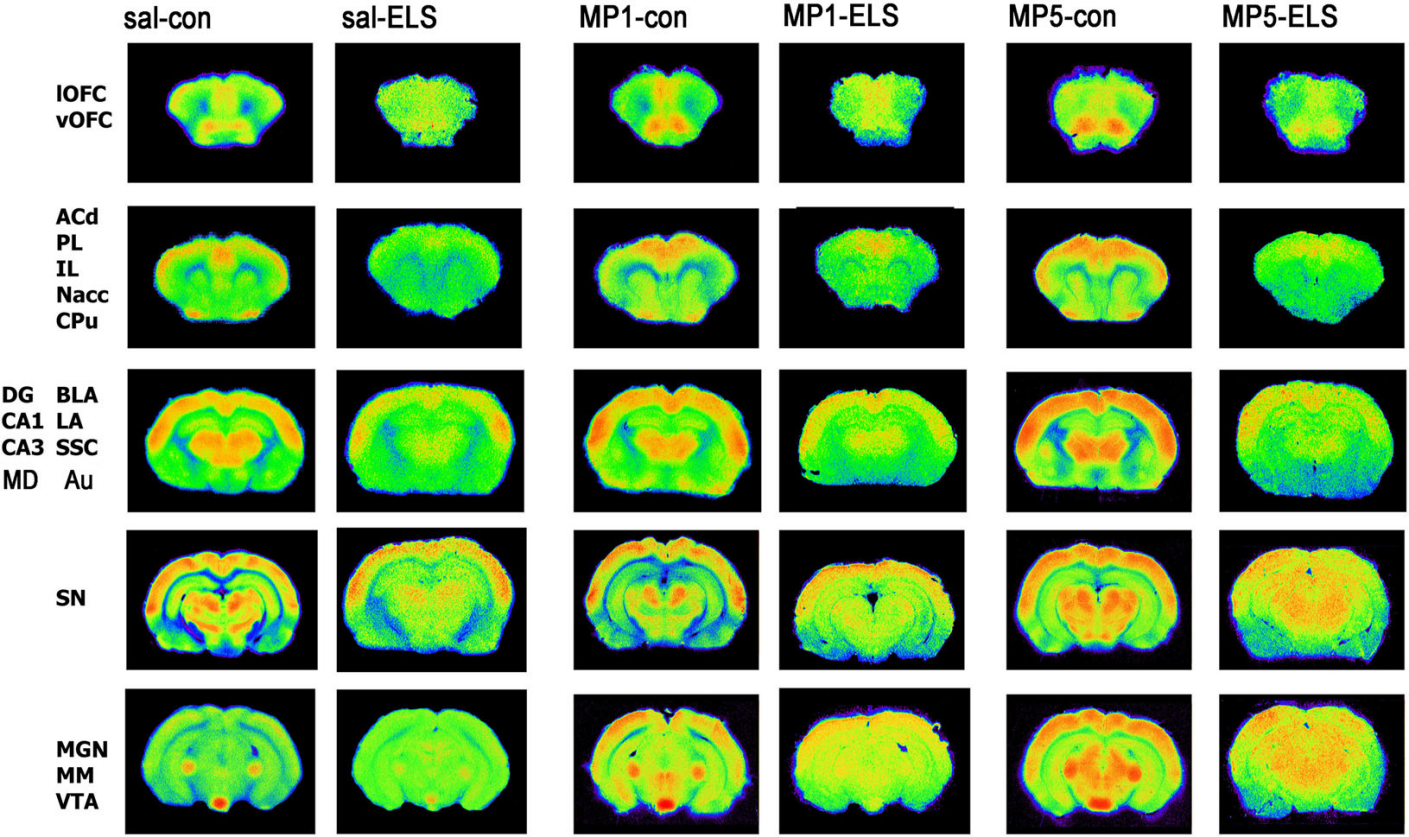
Pseudocolor images of frontal sections indicating 2-FDG uptake as measurement comparing metabolic brain activity in the CON and ELS group under vehicle and MP treatment conditions

High metabolic activities were found in the prefrontal ACd, PL and OFC, as well as in sensory cortices, such as the SSC and Au and also in the subcortical CPu, Nacc, thalamic MD and hippocampal DG. Lower activities were observed in the prefrontal IL, the hippocampal CA1 and CA3 and in the amygdala (BLA, LA) (Fig. 7).

Two-way ANOVA revealed main effects for REAR in all analyzed regions, except the CA1 and CA3. A main effect of PHARM was found for the lOFC (*F*_2,24_ = 3.418), ACd (*F*_2,24_ = 3.774), MD (*F*_2,24_ = 4.551), SN (*F*_2,24_ = 16.17) and VTA (*F*_2,24_ = 6.829). An interaction of REAR × PHARM was found for the Nacc (*F*_2,24_ = 4.217) and SN (*F*_2,24_ = 7.486).

#### Effects of ELS on metabolic brain activity

**Fig. 8:**
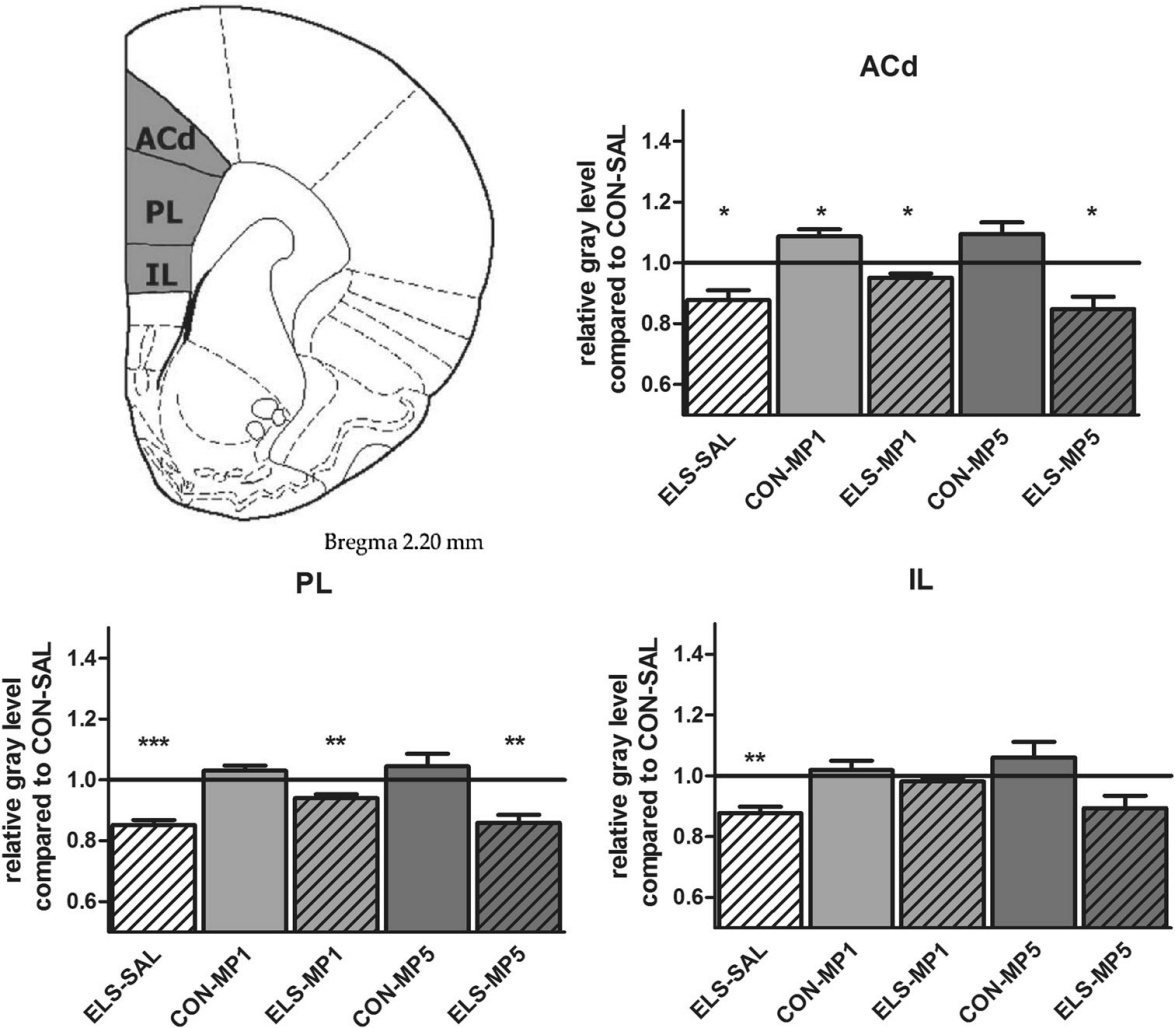
Quantitative analysis of metabolic activity in mPFC regions under vehicle and MP treatment conditions related to the CON-SAL group (*black line*)

**Fig. 9:**
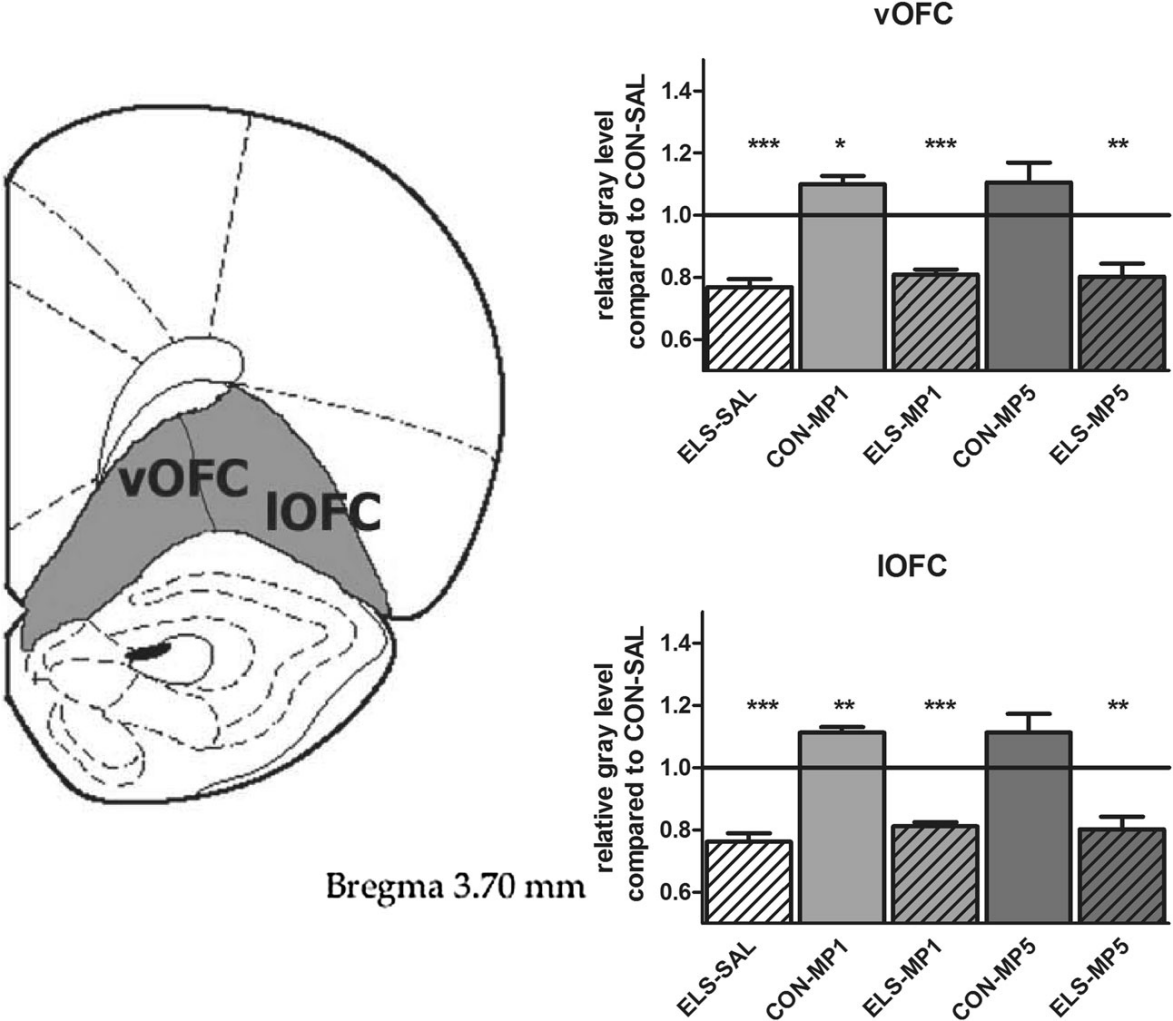
Quantitative analysis of metabolic activity in the ventral and lateral orbitofrontal cortex (OFC) under vehicle and MP treatment conditions in relation to the CON-SAL group (*black line*)

**Fig. 10:**
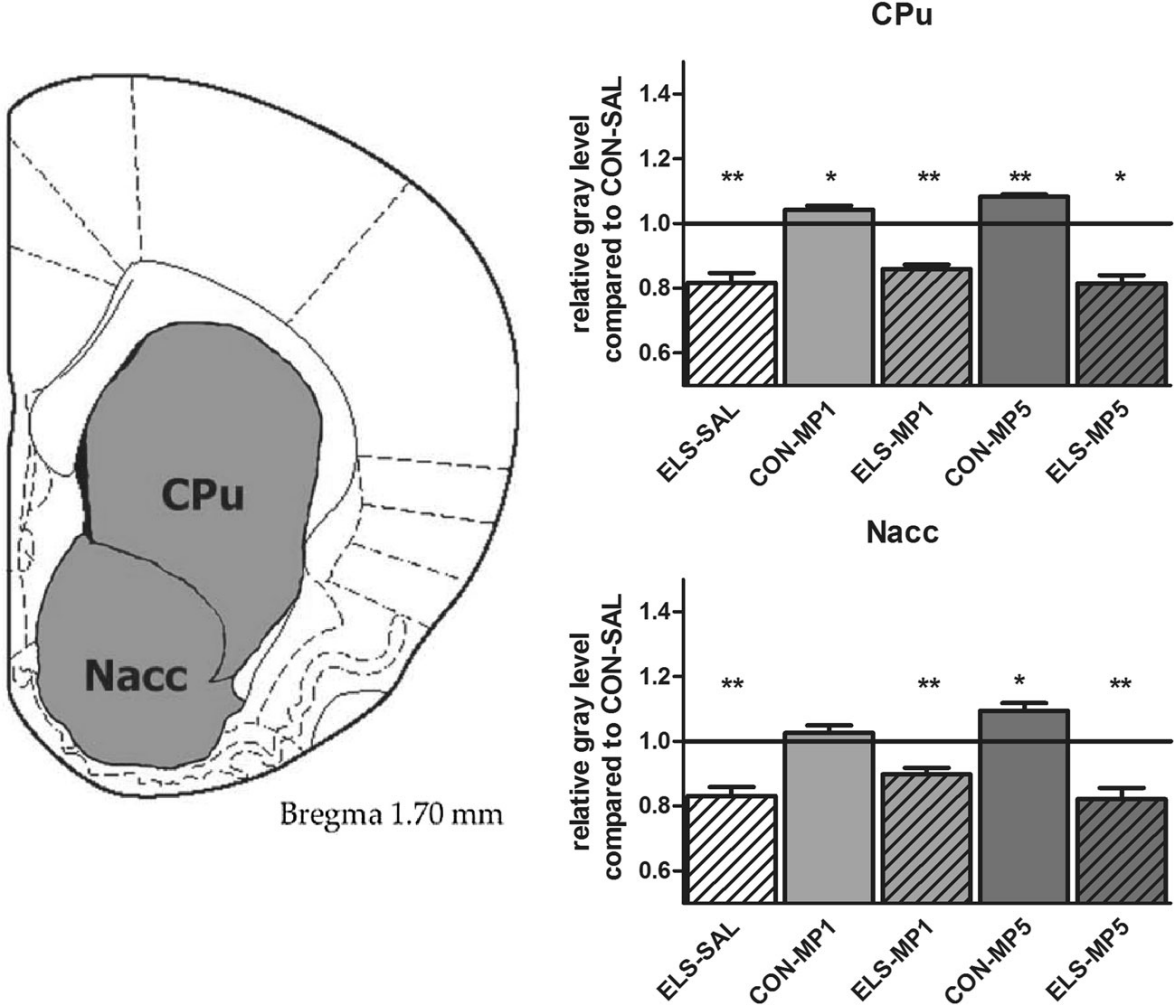
Quantitative analysis of metabolic activity in the striatal CPu and Nacc under vehicle and MP treatment conditions in relation to the CON-SAL group (*black line*)

**Fig. 11:**
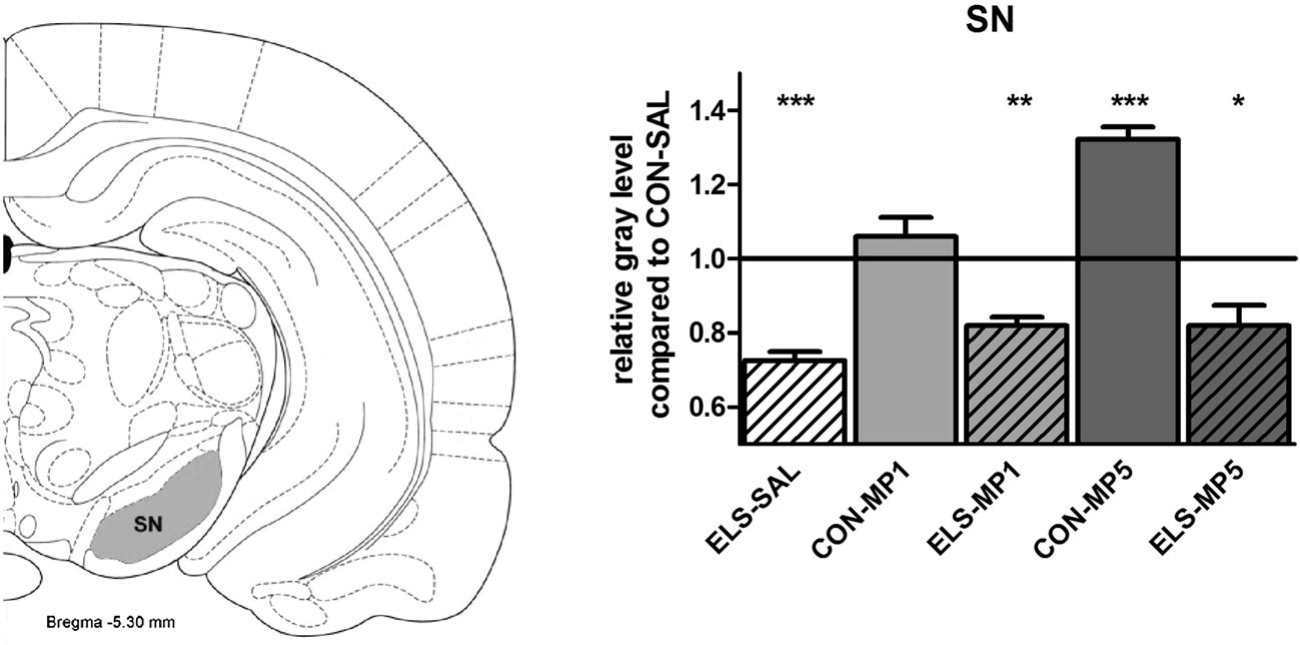
Quantitative analysis of metabolic activity in the SN under vehicle and MP treatment conditions in relation to the CON-SAL group (*black line*)

**Fig. 12:**
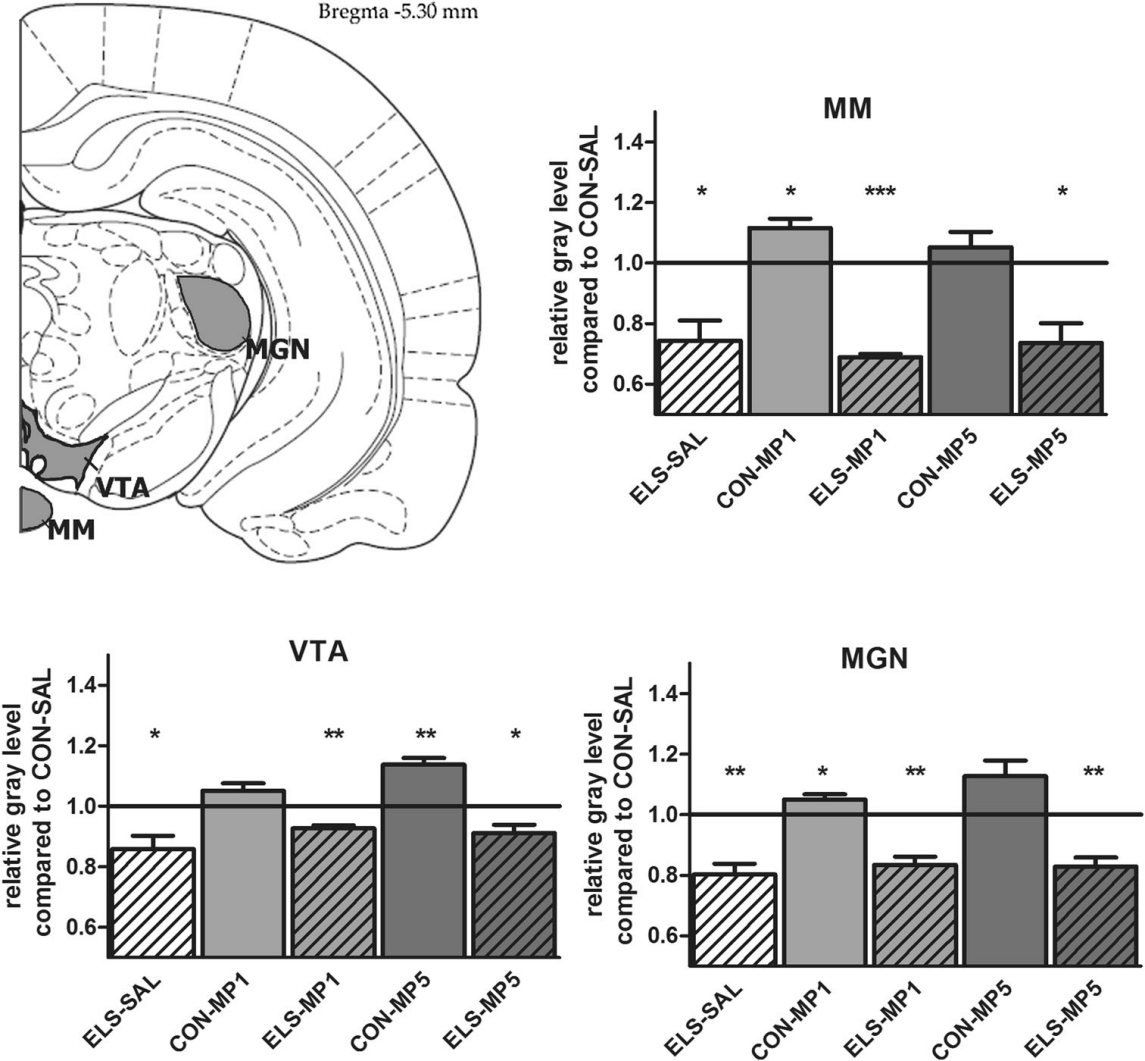
Quantitative analysis of metabolic activity in the MGN, VTA and MM under vehicle and MP treatment conditions in relation to the CON-SAL group (*black line*)

**Fig. 13:**
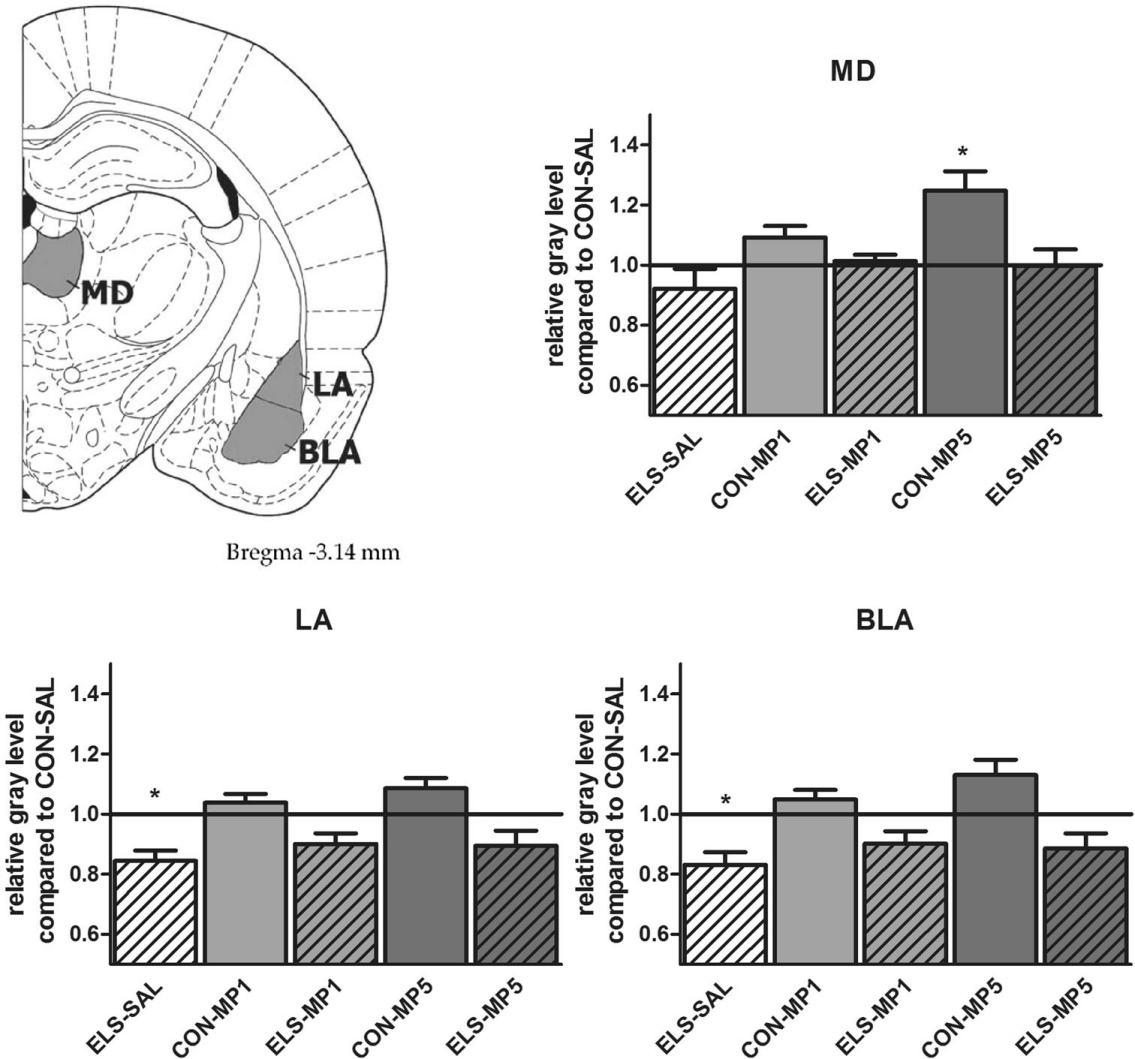
Quantitative analysis of metabolic activity in the MD, LA and BLA under vehicle and MP treatment conditions in relation to the CON-SAL group (*black line*)

**Fig. 14:**
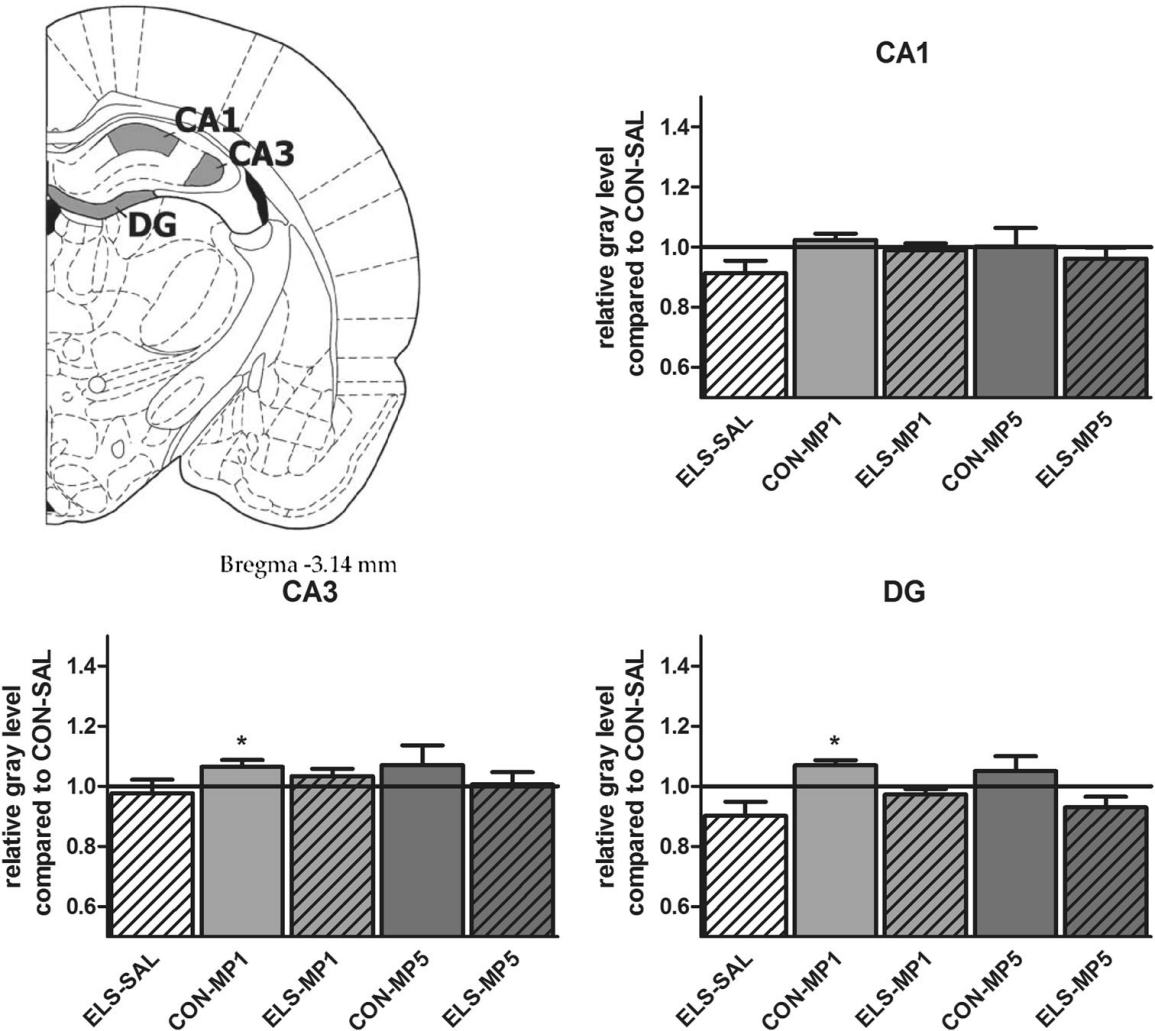
Quantitative analysis of metabolic activity in the hippocampal CA1, CA3 and DG under vehicle and MP treatment conditions in relation to the CON-SAL group (*black line*)

**Fig. 15:**
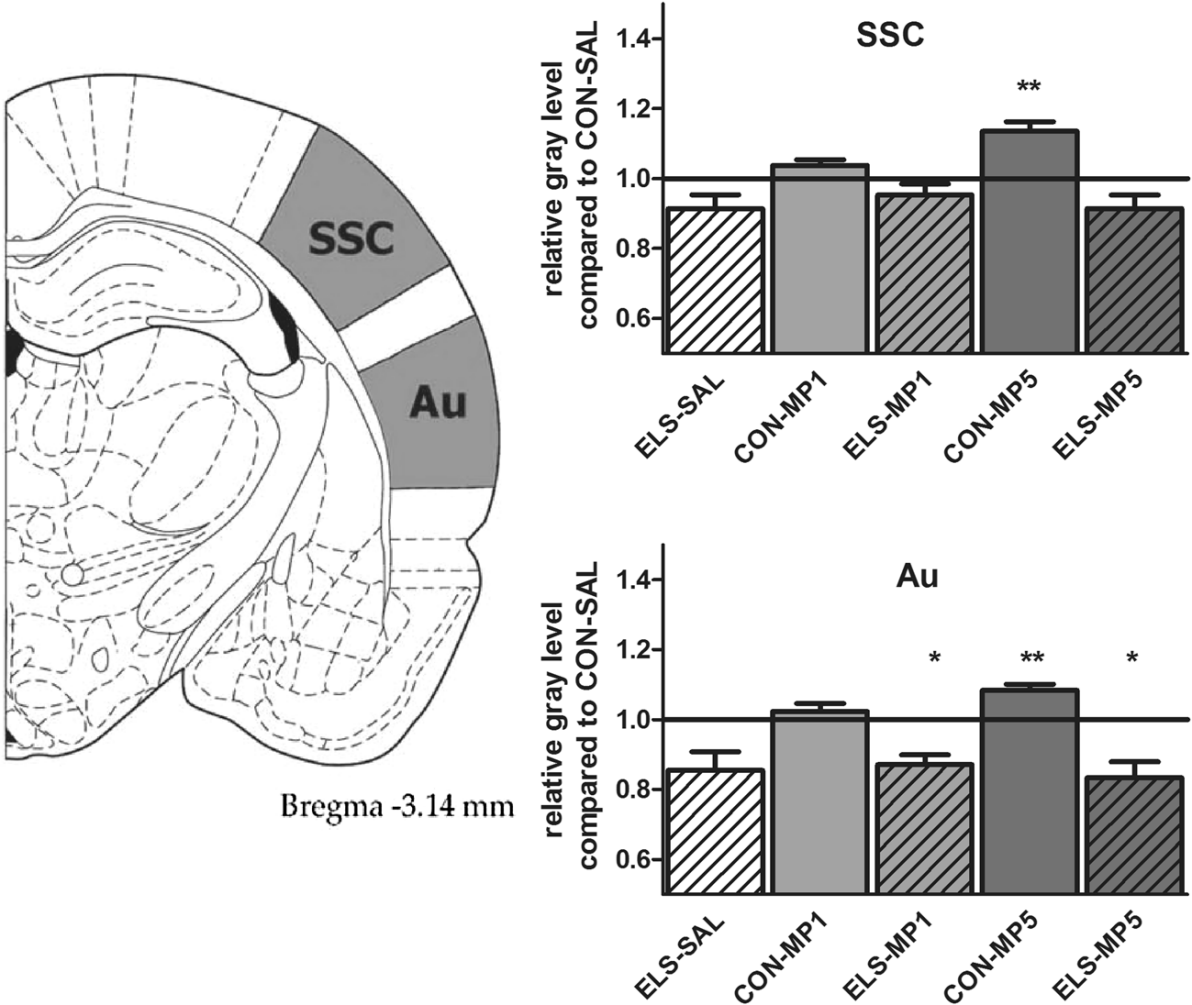
Quantitative analysis of metabolic activity in the Au and SSC under vehicle and MP treatment conditions in relation to the CON-SAL group (*black line*)

Post hoc test revealed that ELS experience resulted in a significant decrease of metabolic activity in the prefrontal ACd (*p* < 0.05), PL (*p* < 0.01), IL (*p* < 0.001) (Fig. 8), vOFC (*p* < 0.001) and lOFC (*p* < 0.001) (Fig. 9), dopaminergic and strongly dopamine innervated regions CPu (*p* < 0.01), Nacc (*p* < 0.01) (Fig. 10), SN (*p* < 0.001) (Fig. 11) and VTA (*p* < 0.05) (Fig. 12), in the limbic BLA (*p* < 0.05), LA (*p* < 0.05) (Fig. 13) and MM (*p* < 0.05) (Fig. 12), and in the auditory MGN (*p* < 0.01) (Fig. 12) when compared to the non-stressed CON-SAL group. No significant differences between the ELS and CON group were observed in MD (Fig. 13), CA1, CA3, DG (Fig. 14), Au and SSC (Fig. 15).

#### Effects of MP treatment on metabolic brain activity

Therapeutic effects of MP treatment were found in the prefrontal IL and in the LA and BLA. MP1 treatment completely normalized metabolic activity in the IL of ELS animals to the levels of the CON-SAL group (Fig. 8) and partly reversed activity in the LA and BLA (Fig. 13), whereas no therapeutic effects could be observed for the other analyzed brain regions.

Similarly, MP5 treatment increased metabolic activity of ELS animals back to control levels in the IL (Fig. 8) as well as in the LA and BLA (Fig. 13). In the CON group, MP1 significantly elevated metabolic activity in the CPu (Fig. 10), CA3, DG (Fig. 14), MM, MGN (Fig. 12), ACd (Fig. 8), and in the vOFC and lOFC (Fig. 9) when compared to the CON-SAL group. In the CON-MP5 group, significantly increased metabolic activity was observed in the CPu, Nacc (Fig. 10), SN (Fig. 11), VTA (Fig. 12), MD (Fig. 13), SSC and Au (Fig. 15) compared to the CON-SAL group.

## Discussion

The present study on *Octodon degus* confirmed our hypothesis that environmental factors such as ELS induce ADHD-like behavioral symptoms, including locomotor hyperactivity and inattentiveness towards an emotionally meaningful acoustic stimulus. Furthermore, ELS induced metabolic hypoactivity in prefronto-limbic brain areas. Finally, we were able to provide evidence that the behavioral and brain metabolic dysfunctions are at least in part caused by abnormal dopamine levels. Treatment of ELS animals with methylphenidate (MP) and thereby elevating dopamine levels ameliorated the behavioral dysfunctions as well as brain hypoactivity in the prefrontal infralimbic cortex and the limbic basolateral and lateral amygdala.

### ELS-induced ADHD-like behavioral symptoms

In line with previous observations (Braun et al. 2003), the present study revealed that ELS exposure results in hyperactive behavior. Moreover, increased exploration in the center area of the open field and increased self-grooming are indicative of reduced impulse control (Colorado et al. 2006; Hall et al. 2000). The reduced running activity and time spent in the quadrant emitting socially attractive conspecific vocalizations is indicative of reduced attentiveness and impaired social interest towards an emotionally relevant acoustic stimulus in ELS animals. In addition, enhanced self-grooming as measured during the modified open field test may be indicative of enhanced sensitivity, impaired behavioral inhibition and reduced anxiety during exposure to novelty and aversive situations (Clement et al. 1995; Clement and Chapouthier 1998; Ramos et al. 1997), i.e., situations, which produce relatively higher inhibition responses such as a session in a brightly illuminated open field (Díaz-Morán et al. 2014; Smolinsky et al. 2009).

### Treatment of ELS animals with methylphenidate (MP) ameliorated ADHD-like behavioral symptoms

The behavioral symptoms observed in our animal model are reminiscent of main features of ADHD, for which the role of the dopaminergic midbrain areas and the connected mesolimbic and nigrostriatal system has been emphasized (Carey et al. 1998; Oades et al. 2005; Russell et al. 1995; Viggiano et al. 2002; Kirley et al. 2002; Solanto 2002). Evidence from research in humans as well as in animal models implicates a dysregulation of the dopaminergic and norepinephrinergic systems in frontostriatal brain circuits in the pathophysiology of ADHD (Biederman and Faraone 2005). Our previous work demonstrated that ELS in degus induces dramatic changes in the density and function of dopaminergic and serotonergic fiber innervation in prefrontal and limbic brain regions (Helmeke et al. 2008; Seidel et al. 2008; Braun et al. 2000; Gos et al. 2006; Poeggel et al. 2003b; Kunzler et al. 2015; Hall et al. 1998; 1999; Jezierski et al. 2007). Thus, the aim was to test the most frequently used drug for treating ADHD methylphenidate (MP) (Sagvolden 2000), a stimulant that acts by blocking dopamine and norepinephrine transporters. Treatment of ELS animals with 1 mg/kg MP completely normalized motor hyperactivity in the standard open field and also attentiveness (reactivity towards conspecific vocalizations) and reduced impulse control (self-grooming) in the modified open field. These findings are in line with studies in behaviorally abnormal, ADHD-type genetic rat strains reporting an amelioration of hyperactivity symptoms under low doses of MP (Kishikawa et al. 2014). On the other hand, our results are somewhat contradictory to a study in which ADHD symptoms were induced by rearing rats in social isolation and impoverished environment, where MP failed to normalize the behavioral dysfunctions (Yates et al. 2012).

It is important to point out that MP treatment evoked quite opposite behavioral effects in ADHD-type ELS and in behaviorally normal CON animals, which indicates that ELS induces changes in dopaminergic functions, e.g., receptors and/or transporter systems. In contrast to the therapeutic effects of low-dose MP in the ELS ADHD-type animals, MP treatment in control animals in general induced hyperactive behavior and reduced attentiveness. Pharmacological studies, which tested the behavioral effects of MP treatment in *behaviorally normal* rodents, also observed typical stimulant effects such as increased locomotor activity (Thanos et al. 2015; Haleem et al. 2015; Berridge et al. 2006), but in contrast to our study improved attention (Bhattacharya et al. 2015).

### ELS-induced metabolic hypoactivity in prefronto-limbic brain regions

The present study is the first to show that  exposure to ELS results in lower metabolic activity in executive/motor pathways including the prefrontal cortical areas OFC, ACd, PL and also the substantia nigra (SN). It is tempting to speculate that ELS hypoactivity of these regions is causally linked to the observed ADHD-like behavioral symptoms, analogous to brain dysfunctions found in ADHD patients (Dahmen et al. 2012; Rubia et al. 2005; Smith et al. 2006).

Additionally, we found that ELS animals display reduced metabolic activity in brain regions mediating impulse control such as the ventral striatum (Nacc) (Feja et al. 2014), the ACd and IL (Tsutsui-Kimura et al. 2016) and also in affective/learning/motivation pathways, including the prefrontal cortex, the VTA, Nacc, the amygdala (BLA, LA) and the mammillary bodies.

The ELS-induced prefronto-limbic/prefrontostriatal hypofunctionality observed in our animal model is in accordance with findings of fMRI studies in ADHD patients, which have shown task-related hypofunction in the mPFC (Casey et al. 1997; Rubia et al. 1999). Compared to healthy controls, individuals with ADHD are also reported to show abnormalities in brain activation during response inhibition, including hypoactivation of the anterior/mid-cingulate cortex (Tamm et al. 2004). ADHD children show a slightly different cognitive profile at 6–10 years of age that was paralleled by a relative lack of or delay in the maturation of ventral frontostriatal circuitry (Durston et al. 2003). Additionally, fMRI studies in ADHD patients revealed that striatal dysfunctions are directly connected with altered motor behavior (Teicher et al. 2000).

### MP treatment partly ameliorated ELS-induced metabolic hypoactivity in prefronto-limbic brain areas

Most of the affected brain areas in ELS animals are regions of the fronto-striatal system, which is modulated by dopaminergic input, and it is believed that dysregulation of dopaminergic modulation in these circuits contributes to the pathophysiology of ADHD (Biederman and Faraone 2005). It is tempting to speculate that ELS impairs the maturation of dopaminergic functions, which might affect metabolic activity in limbic and prefrontal brain circuits. To test whether elevating dopamine level “normalizes” metabolic brain activity, we treated young ELS animals with methylphenidate. Indeed, a therapeutic effect of MP treatment was observed, i.e., metabolic activity was upregulated in the IL as well as in the LA and BLA of MP-treated ELS animals. The IL is involved in the regulation of attentiveness and behavioral flexibility (Oualian and Gisquet-Verrier 2010; Ragozzino et al. 1999) and the IL–amygdala pathway in the regulation of emotional behavior (Sotres-Bayon and Quirk 2010; Biederman and Faraone 2005; Cunningham et al. 2002), which at least in part matches the behavioral effects of MP treatment, in particular the normalization of attentiveness and reactivity towards an emotionally relevant tone stimulus.

Taken together our study revealed that ELS as environmental challenge induces metabolic hypoactivity in brain pathways mediating attentiveness, which are paralleled by behavioral traits reminiscent of human ADHD. These dysfunctions are due to dopaminergic dysfunctions as they are ameliorated by MP treatment. Our results underline the importance to consider an adverse childhood environment as an important factor in the etiology of ADHD-like behavioral abnormalities.

## Abbreviations

PND: Postnatal day
DA: Dopamine
MP: Methylphenidate
SAL: Saline
i.p.: Intraperitoneal
CON: Unstressed control group
ELS: Early life stress
REAR: Rearing conditions (control, early stress)
PHARM: Pharmacological treatment
HEM: Hemisphere
2-FDG: (^14^C)-2-Fluoro-deoxyglucose autoradiography (2FDG)
OF: Open field
ACd: Dorsal anterior cingulate cortex
Au: Auditory cortex
BLA: Basolateral amygdaloid nucleus
CPu: Striatum
DG: Hippocampus-dentate gyrus
CA1: Hippocampus-CA1
LA: Lateral amygdaloid nucleus
MGN: Medial geniculate nucleus
MM: Medial mammillary nuclei
Nacc: Nucleus accumbens
SN: Substantia nigra
SSC: Somatosensory cortex
MD: Mediodorsal thalamus
